# Dataset of global cities’ congestion level and urban spatial pattern

**DOI:** 10.1016/j.dib.2022.108440

**Published:** 2022-07-04

**Authors:** Jiewei Li, Ming Lu, Tianyi Lu

**Affiliations:** a School of Economics and Management, Shanghai Maritime University, Shanghai 201306, China; b Department of Economics, Antai College of Economics and Management, Shanghai Jiao Tong. University, Shanghai 200030, China; c School of Economics, Zhejiang University, Hangzhou 310000, China

**Keywords:** Compact city, Urban spatial pattern, Congestion, Skyscraper index

## Abstract

This dataset includes many indexes of global cities. The variables of congestion level, skyscraper index, whether a city was bombed in WWII (World War II), and global cities’ population are key variables. (1) The congestion level data were collected from TOMTOM company. The congestion level data include five indexes which are “Congestion level”, “Morning peak Congestion level”, “Evening peak Congestion level”, “Highways Congestion level”, “Non-highways Congestion level” in 2004, but only include two indexes in 2020 which are “Time lost per year” and “Congestion level”. (2) The data of skyscraper index calculated using the data of building height from the Council on Tall Buildings and Urban Habitat, from which we can obtain accurate data on the number of buildings taller than 150 m. With these data, we constructed an index of skyscrapers taller than 150 m in a city. A building receives a score of 1.5 if it is taller than 150 m and shorter than 200 m, 2.0 if it is between 200 m and 300 m, and so on. Then, we summed the scores for skyscrapers in the city as the “skyscraper index” of the city. (3) The data of whether a city was bombed in WWII is dummy variable, if the urban area of a city was bombed in WWII, it is 1, and 0 otherwise. The authors consulted various historical files and determined the value. (4) The data of global cities’ population, as well as the area and density of the city, are on the city-level, and were collected from the website of the cities or countries’ statistics department. These indicators are good measures of the level of congestion, urban spatial structure, IV(instrumental variable) for urban spatial structure, and urban population in global cities, and can be reused in other analysis.

## Specifications Table

**Table:** 

Subject	Economics, Econometrics and Finance
Specific subject area	Regional science and urban economics
Type of data	Table
How the data were acquired	Collected manually from the website, book, paper and other files.It can be analyzed by Stata, excel and other software.
Data format	Raw: csvAnalyzed: txt
Description of data collection	The data of skyscraper index was Data from the Council on Tall Buildings and Urban Habitat, from which we can obtain accurate data on the number of buildings taller than 150 m. Using these data, we constructed an index of skyscrapers taller than 150 m in a city. A building receives a score of 1.5 if it is taller than 150 m and shorter than 200 m, 2.0 if it is between 200 m and 300 m, and so on. Then, we summed the scores for skyscrapers in the city as the “skyscraper index” of the city.The data of whether a city was bombed in WWII is dummy variable, if the city was bombed in WWII, it is 1, and 0 otherwise.The density is obtained by dividing population by area.Data of other variables are not processed.
Data source location	Height of skyscraper: the Council on Tall Buildings and Urban Habitat, https://www.skyscrapercenter.com/cities.Congestion level: TOMTOM, https://www.tomtom.com/en_gb/traffic-index/.Whether a city was bombed in WWII: We determined whether a city was bombed during World War II by comparing various kinds of data. The major documents are listed as follows:Books:L. Hart, History of the Second World War. New York: Putnam Publishing Group, 1971.J. Keegan, The Second World War. New York: Viking Press, 1990.Papers:G.Y. Gao, D.T. Wang, Y. Che, Impact of Historical Conflict on FDI Location and Performance: Japanese Investment in China, J. of Int. Bus. Stud. 49 (2018) 1060-1080.D.R. Davis, D.E. Weinstein, Bones, Bombs, and Break Points: The Geography of Economy Activity, Am. Econ. Rev. 92 (2002) 1269-1289.Records:The introduction of every city in Baidu (https://www.baidu.com/) and Wikipedia (http://www.Wikipedia.org/)The introduction of every battle in WWII in Baidu (https://www.baidu.com/) and Wikipedia (http://www.Wikipedia.org/)Documentaries:“Apocalypse” (https://www.natgeotv.com/asia/apocalypse-the-second-world-war)
Data accessibility	https://doi.org/10.6084/m9.figshare.20146844
Related research article	*J. Li, M. Lu, T. Lu, Constructing Compact Cities: How Urban Regeneration Can Enhance Growth and Relieve Congestion, Econ. Modelling. 113 (2022) 1-10.*https://doi.org/10.1016/j.econmod.2022.105828

## Value of the Data

• These indicators are good measures of the level of congestion, population of a city, and urban spatial structure etc. First of all, there are few congestion indexes which can cover a great number of cities all over the world. However, the congestion index used in this paper were collected and calculated by TOMTOM, a famous navigation and mobile location services company, which provided congestion data of 448 cities. Since the data were collected and calculated by the same company, this indicator can be compared well with each other in cross-section. Secondly, the population statistics varies from country to country, like city-level, MSA(Metropolitan Statistical Areas)-level etc.. We tried our best to collected the population of 511 cities around year 2013. The urban spatial structure indexes of global cities were also calculated in the same standard, so they can be compared with each other.
• The indicator of “Whether a city was bombed in WWII” is a good IV (instrumental variable) for urban spatial pattern [1]. It was found in many countries, that the buildings of an area (or a city) will be higher if the area was bombed during WWII. And “Whether a city was bombed in WWII” is a good IV (instrumental variable) for urban spatial pattern used in some researches. However, there are few data sets of “Whether a city was bombed in WWII” for global cities. The data set provided covers 511 cities all over the world.
• If a researcher wants to do the research on regional science and urban economics, especially urban spatial pattern and congestion [2], he/she can use these data.
• The researcher who wants to do global urban study can merge the date with other city-level variables and do the research.

## Data Description

• The CCCdata1.csv contains 513 city observations, with the congestion index of 2019 and the skyscraper index of 2020.

**Table:** 

variables	Description of variables
City	City name.
Country	The country this city belongs to.
Popcity	Population of the city (person).
areacity	Area of the city (km^2^).
densitycity	Population density of the city (person/km^2^).
congestion2019	Congestion level of the city in year 2019(%), measuring travel times during the whole day and compares these with measured travel times during non-congested periods (Free Flow conditions).
bombed	Whether the city was bombed during WWII (bombed=1, 0 otherwise).
SI	The skyscraper index of the city which represents the urban spatial pattern.

• The CCCdata2.csv contains 82 city observations, with the congestion index of 2014 and the skyscraper index of 2014.

**Table:** 

variables	Description of variables
City	City name.
Country	The country this city belongs to.
congestionlevel	Average congestion level of the city in year 2014(%), measuring travel times during the whole day and during peak periods and compares these with measured travel times during non-congested periods (Free Flow conditions).
morningpeak	Congestion level of the city during morning peak (%).
eveningpeak	Average congestion level of the city during evening peak (%).
highways	Average congestion level of the city in highways (%).
nonhighways	Average congestion level of the city in nonhighways (%).
indexofbuildingdensity150800	The skyscraper index of the city which represents the urban spatial pattern.
indexofbuildingdensity150800mixr	The skyscraper index of the city with only mixed-use buildings.
indexofbuildingdensity150800resi	The skyscraper index of the city with only residential buildings.
bombed	Whether the city was bombed during WWII (bombed=1, 0 otherwise).
population	Population of the city (person).

• The CCCSTATAcode.txt is the code for Stata.

## 2 Experimental Design, Materials and Methods

(1) The variable of skyscraper index: the raw data came from the Council on Tall Buildings and Urban Habitat, from which we can obtain accurate data on the number of buildings taller than 150 m. With these data, we constructed an index of skyscrapers taller than 150 m in a city. A building receives a score of 1.5 if it is taller than 150 m and shorter than 200 m, 2.0 if it is between 200 m and 300 m, and so on. Then, we summed the scores for skyscrapers in the city as the “skyscraper index” of the city. The variables of indexofbuildingdensity150800mixr and indexofbuildingdensity150800resi were calculated using the same method, but only with the mixed-use buildings and residential buildings.
(2) The variable of densitycity: The city population density was obtained by dividing the city population by the area of the city.
(3) The variable of whether a city was bombed during WWII: We compared various kinds of data and verified with each other to determine whether the urban area of a city was bombed during World War II. If the urban area of a city was bombed during World War II, the indicator is 1, otherwise it is 0.
(4) Other variables: Other variables are raw data without calculation.

## CRediT Author Statement

**Li Jiewei:** Conceptualization, Collecting Data and Editing, Methodology, Software, Supervision; **Lu Ming:** Conceptualization, Methodology, Supervision; **Lu Tianyi:** Conceptualization.

## Role of the Funding Source

The funding source has nothing to do with this research except paying the submission fee.

## Declaration of Competing Interest

The authors declare that they have no known competing financial interests or personal relationships that could have appeared to influence the work reported in this paper.

## Data Availability

Constructing compact cities: How urban regeneration can enhance growth (Original data) (figshare repository).
